# Miro1 overexpression protects against α-synuclein-induced mitochondrial loss in neuronal culture

**DOI:** 10.1186/2193-1801-4-S1-P41

**Published:** 2015-06-12

**Authors:** Dzhamilja Safiulina, Vinay Choubey, Miriam Ann Hickey, Allen Kaasik

**Affiliations:** Department of Pharmacology, Institute of Biomedicine and Translational Medicine, University of Tartu, Estonia

**Keywords:** mitochondria, autophagy, parkinson’s disease

Mitophagy is a selective degradation of mitochondria that promotes the turnover of mitochondria and prevents the accumulation of damaged organelles. Pink1 and Parkin proteins are crucial in the removal of damaged mitochondria. Mutations in the corresponding PINK1 and PARK2 genes are associated with Parkinson’s disease (PD), and mitochondrial impairments are central to PD pathogenesis. Pink1 has been shown to interact with the atypical Rho GTPases Miro, the outer mitochondrial membrane proteins involved in mitochondrial trafficking. Miro1 is degraded shortly after mitochondrial damage in a Parkin-dependent manner. α-synuclein is a major component of Lewy bodies, the characteristic cellular inclusions in PD. Mutations of α-synuclein, including A53T, are linked to familial PD. α-synuclein has been shown to bind to the mitochondrial membrane, and increased membrane-bound α-synuclein in PD contributes to the functional disturbance of mitochondria. Overexpression of A53T-mutated α-synuclein has been shown to induce mitophagy in vitro and in vivo. We hypothesized that Miro1 function is disturbed in an α-synuclein (A53T)-overexpressing model, and the aim of this study was to elucidate the involvement of Miro1 in α-synuclein-induced mitophagy. We have found that α-synuclein is one component of the Miro1 interactome. Moreover, co-expression of Miro1 restored mitochondrial length and density in primary neuronal culture overexpressing A53T-mutated α-synuclein. Miro1 overexpression did not change the basal mitophagy, but decreased significantly α-synuclein-induced mitochondrial removal. Together, our results suggest that Miro and α-synuclein may interact in the mitophagic pathway.

